# Recent advances in the management of ventricular tachyarrhythmias

**DOI:** 10.12688/f1000research.11202.1

**Published:** 2017-06-29

**Authors:** Syeda Atiqa Batul, Brian Olshansky, John D. Fisher, Rakesh Gopinathannair

**Affiliations:** 1 Arrhythmia Division, Montefiore Medical Center, Bronx, New York, USA; 2 University of Iowa Hospitals and Clinics, Iowa City, USA; 3 Division of Cardiovascular Medicine, University of Louisville School of Medicine, Louisville, USA

**Keywords:** Ventricular Arrhythmias, cardiomyopathy, catheter ablation, implantable cardioverter-defibrillator

## Abstract

Ventricular arrhythmias are an important cause of cardiovascular morbidity and mortality, particularly in those with structural heart disease, inherited cardiomyopathies, and channelopathies. The goals of ventricular arrhythmia management include symptom relief, improving quality of life, reducing implantable cardioverter defibrillator shocks, preventing deterioration of left ventricular function, reducing risk of arrhythmic death, and potentially improving overall survival. Guideline-directed medical therapy and implantable cardioverter defibrillator implantation remain the mainstay of therapy to prevent sudden cardiac death in patients with ventricular arrhythmias in the setting of structural heart disease. Recent advances in imaging modalities and commercial availability of genetic testing panels have enhanced our mechanistic understanding of the disease processes and, along with significant progress in catheter-based ablative therapies, have enabled a tailored and more effective management of drug-refractory ventricular arrhythmias. Several gaps in our knowledge remain and require further research. In this article, we review the recent advances in the diagnosis and management of ventricular arrhythmias.

## Introduction

Sudden cardiac death claims 350,000 to 400,000 lives annually in the US ^1^. Ventricular fibrillation (VF) or pulseless ventricular tachycardia (VT) accounts for 25 to 36% of witnessed cardiac arrests at home and 38 to 79% of witnessed cardiac arrests in public ^2^. Ventricular arrhythmias (VAs) represent a broad spectrum spanning single ectopic beats to sustained VT and VF. These arrhythmias encompass clinical conditions ranging from benign to life threatening. Sustained VAs are mostly associated with structural or ischemic heart disease (60 to 80%) followed by channelopathies and idiopathic VAs (VA in the absence of structural heart disease) ^3, 4^. Although life-threatening VAs are greatest in those with known coronary artery disease, myocardial infarction, and depressed ejection fraction, a considerable number of patients with non-ischemic cardiomyopathy (NICM) experience fatal events due to VT or VF as well ^5^.

Different forms of VAs can coexist in the same patient, can be isolated or frequent, and have differing mechanisms. Premature ventricular contractions are the most common and are generally considered benign. Non-sustained VT is at least three consecutive ventricular beats at a rate of at least 100 beats per minute; if VT exceeds 30 seconds or is hemodynamically unstable, it is considered “sustained”. VT can be regular or irregular in rate and morphology. Sustained monomorphic VT has the same activation sequence from beat to beat and generally occurs because of a stable re-entry circuit in patients with structural heart disease or because of automaticity in idiopathic VTs. The 12-lead electrocardiogram (ECG) provides an approximation of the exit site. On the other hand, polymorphic VT that often degenerates into VF is more often seen in acute myocardial ischemia or infarction as well as with several genetically mediated syndromes ^6^.

The goals of management of VAs include symptom relief (including syncope, worsening heart failure, and ischemic chest pain), improving quality of life, reducing implantable cardioverter defibrillator (ICD) shocks, preventing deterioration of left ventricular function, reducing risk of arrhythmic death, and improving overall survival ^7– 10^. Treatment of VAs should take into account the underlying medical conditions, the cardiac disorders, the presence of heart failure, the cause for the arrhythmias, consequences of the VAs, and the risks and benefits of the therapeutic pharmacological or invasive strategy ^6, 8, 9^.

ICDs are the mainstay of therapy to reduce the risk of sudden cardiac death due to VT and VF. Despite the mortality benefit gained from ICD therapy, electrical shocks from the device as well as unopposed and unnecessary right ventricular pacing should be monitored for and appropriately addressed ^7, 11– 13^. According to current data, both anti-arrhythmic drugs and catheter ablation may reduce the recurrence of VAs without offering any survival benefit ^10^. Recurrent ventricular tachyarrhythmias and ICD shocks are the major indication for anti-arrhythmic drug therapy, but these medications can also be pro-arrhythmic ^13^. Catheter ablation has evolved as a promising therapy to reduce the risk of VT recurrence; it is superior to medical therapy alone, according to clinical trials ^14– 16^. Despite improved mechanistic understanding and progress in catheter ablation technology that has led to effective treatment of focal and idiopathic VT, the long-term success rates for VT ablation remain modest in both ischemic and non-ischemic cardiomyopathies ^17^.

Considerable research and clinical effort in recent years have been focused on the development of diagnostic modalities and imaging tools to identify the arrhythmogenic substrate responsible for VT (focal or scar), genetic screening for markers of channelopathies, and superior mapping and ablation technologies. These advances have allowed us to tailor our approach to VA management on the basis of the underlying etiology with higher efficacy ^6, 8, 9^.

In this review, we aim to provide an update on the newer advances in the management of VAs.

## Update on diagnostic and non-invasive imaging modalities

For over 100 years, cardiologists relied on the ECG, monitoring strategies, and limited intracardiac electrodes to diagnose VAs. With mechanisms of VT now better defined in structural heart disease, non-invasive imaging modalities—including echocardiography, cardiac computed tomography (CT), cardiac magnetic resonance imaging (MRI) (CMR), and nuclear studies—have become an integral part of the diagnostic and management strategy. The information derived from these tests not only is important to identify those with structural heart disease, assess left and right ventricular function, and risk-stratify those at highest risk of inducible VT/VF on the basis of scar size in order to select the patients who would derive the most benefit from an ICD but also serves as a guide for planning ablation procedures ^8, 18, 19^. The 2015 European Society of Cardiology Guidelines have extended a class IIa recommendation to perform CMR or CT in patients where echocardiography fails to provide accurate assessment of ventricular function or underlying structural changes that may be arrhythmogenic, such as the scar size, distribution, and transmurality ^8^.

Although there are no large randomized controlled trials, multiple small studies have reported a potential benefit of obtaining pre-procedural imaging such as delayed enhancement CMR (DE-MRI) and incorporating the information into intracardiac mapping systems ^20^. Piers *et al*. ^21^ described the characteristics of critical isthmus sites in 44 patients with ischemic cardiomyopathy and NICM undergoing VT ablation with pre-procedural CMR. The authors found the critical VT isthmus sites to be in close proximity to areas of higher (>75%) scar transmurality and scar border zones as noted in CMR.

Contrast-enhanced MRI- and CT-derived scar data have been shown to correlate well with electroanatomic mapping (EAM) reconstructions. In a recent study by Esposito *et al*. ^22^, CT with delayed enhancement provided a detailed three-dimensional (3D) scar characterization with good sensitivity (76%) and specificity (86%) and a very high negative predictive value (95%) when correlated with voltage on EAM. Whereas EAM may be unable to quantify the non-transmural parts of the scar, information obtained from pre-procedural CMR may help in these instances.

Substrate characterization with CMR is also very useful in hypertrophic cardiomyopathy (HCM), arrhythmogenic right ventricular dysplasia (ARVD), and acquired immunological and inflammatory conditions (for example, sarcoidosis) ^22– 28^. A minority of patients with sustained left bundle branch block and inferior axis morphology VT, resembling idiopathic outflow tract VT, may have early concealed ARVD. CMR can help differentiate true idiopathic VT from early-stage ARVD or sarcoidosis and change the clinical approach for these patients ^29^. CMR may be of particular value in those with abnormal resting ECG (for example, right precordial T wave inversion in V1–V3).

Contrast CT has the added advantage of providing good visualization of coronary arteries. Lipomatous metaplasia (myocardial fat deposition) along with fibrosis in areas of chronic myocardial infarction has been observed in patients with ischemic heart disease on contrast CT and has been associated with fractionated electrograms and critical VT circuits ^30^. In patients with VT due to a local (myocarditis) or systemic (for example, sarcoidosis) inflammatory condition, fluorine-18 fluoro-2-deoxyglucose (18-FDG) positron emission tomography (PET/CT) can identify patients who may benefit from early immunosuppressive therapy versus catheter ablation ^31^.

The future of non-invasive mapping of VAs will focus on the creation of image-based simulation to estimate ablation targets in VAs, integration of multi-lead electrocardiography to construct computerized activation maps, and endo-epicardial and perhaps also mid-myocardial signal-intensity mapping that will identify the pathological substrates and VT exit sites which can be targeted more efficaciously ^32– 34^.

## Advances in channelopathies and inherited cardiomyopathies

Up to half of the families of sudden cardiac arrest victims below 50 years of age are carriers of cardiac genetic channelopathies or hereditary cardiomyopathies. The 2015 Expert Consensus Statement from the Heart Rhythm Society recommends that patients and first-degree relatives with diagnosed or suspected inherited cardiovascular disease as a potential cause of sudden cardiac arrest be evaluated in a dedicated clinic with appropriately trained staff (class I) ^9, 35^.

Extended molecular gene panels now can be performed at appropriate centers at a low cost, allowing early identification of a channelopathy or inherited cardiomyopathy. This, in turn, allows risk stratification of sudden cardiac death and introduction of appropriately timed therapy (lifestyle changes, pharmacologic therapy versus ICD implantation or catheter ablation or both ( Table 1) ^9, 35– 37^.

**Table 1.** Heart Rhythm Society/European Society of Cardiology recommendations for the diagnosis and treatment of genetic arrhythmia syndrome and inherited cardiomyopathies.

Genetic arrhythmia syndrome	Associated gene and ion channel affected	Recommendations for diagnosis	Recommendations for treatment
LQTS	• Associated with mutations in at least 15 genes • Three main genes: *KCNQ1* (LQTS1), *KNCH2* (LQTS2), and *SCN5A* (LQTS3) (75% of cases) • *IKr* (LQTS1), *IKs* (LQTS2), *INa* (LQTS3), *IK1* (LQTS7), and *ICa-L* (LQTS8) • AD LQTS with QT prolongation alone (Romano-Ward syndrome) LQTS 1–6 • AD LQTS7 (Anderson-Tawil syndrome): facial dysmorphism and hyper/hypokalemic periodic paralysis • AD LQTS8 (Timothy syndrome) prolonged QT, facial dysmorphism, cardiac malformations, and autism • Autosomal recessive LQTS (Jervell and Lange-Nielson syndrome) extremely prolonged QT interval with congenital deafness	• QTc ≥ 480 ms in repeated 12- lead ECGs or LQTS risk score >3 (class I) • Confirmed pathogenic LQTS mutations (class I) • QTc ≥ 460 ms with unexplained syncopal episode in the absence of secondary causes of QT prolongation	• Avoid QT prolonging agents, correct electrolytes, and avoid genotype- specific triggers (for example, strenuous exercise for LQTS1 and loud noise for LQTS2) • BBs are recommended for patients with clinical diagnosis (class I) and carriers of causative mutation and normal QTc (class IIa) • ICD should be implanted in patients with prior SCA (class I), syncope and/or VT while on adequately dosed BBs (class IIa) or considered in addition to BBs in asymptomatic carriers of *KCNH2* or *SCN5A* mutations and a QTc > 500 ms • LCSD can be attempted in patients with ineffective or poorly tolerated BB therapy, contraindication to ICD, or incessant VT, multiple ICD shocks (class IIa) • Sodium channel blockers (Mexiletine, Flecainide, Ranolazine) may be used as adjunctive therapy in LQTS3 with QTc >500 ms • Invasive EPS and PVS is not recommended for risk stratification (class III)
SQTS	• *KCNH2* (SQT1), *KCNQ1* (SQT2), and *KCNJ2* (SQT3) • *IKr* (SQT1), *IKs* (SQT2), and *IK1* (SQT3)	• QTc ≤ 340 ms on ECG (class I) • QTc ≤ 360 ms and confirmed pathogenic mutation, family history of SQTS, family history of SCD <40 years, survival from VT/VF in the absence of heart disease (class IIa)	• ICD implant is recommended in patients with spontaneous sustained VT or SCA (class I) • Quinidine or sotalol are recommended in patients who refuse or have a contraindication to ICD or in asymptomatic patients with SQTS and a family history of SCD (class IIb) • Invasive EPS and PVS are not recommended for risk stratification (class III).
BrS	• Inherited AD • 12 genetic mutations identified • *SCN5A* and *CACN1Ac* (account for more than 5% of positively genotyped patients) • Genetic screening does not affect treatment or prognosis.	• ST-segment elevation with type 1 morphology ≥2 mm elevation in one or more right precordial leads V1 and/or V2 positioned in the second, third, or fourth intercostal space, occurring either spontaneously or after provocative drug test with intravenous administration of sodium channel blockers such as ajmaline, flecainide, and procainamide (class I)	• Lifestyle changes: Patients should avoid drugs that may induce ST elevation, excessive alcohol consumption, and seek prompt treatment of fever (class I) • ICD implantation is recommended in survivors of SCA and/or spontaneous sustained VT or both (class I) or in patients with spontaneous Brugada pattern and history of syncope (class IIa) or patients with diagnosis of BrS who develop VF during PVS with two or three extra stimuli at two sites (class IIb) • Quinidine or isoproterenol to be considered in electrical storm (class IIa). Quinidine should be considered in patients with BrS who refuse or have a contraindication to ICD implantation (class IIa) • Catheter ablation may be considered in patients with repeated ICD shocks and electrical storm (class IIb).
ERS	• Polygenic • Role of genetic screening is not clear • *IK-ATP* (ERS1, 5), *ICa* (ERS2-4), and *INa* (ERS6) • No clear familial transmission	• Asymptomatic early repolarization pattern is common in general population. • ERS diagnosed only in patients with a pattern and documented idiopathic VF or polymorphic VT	• Insufficient evidence to make definitive recommendations
CPVT	• Dominant variant: ryanodine receptor gene ( *RyR2*) • Rare recessive variant: cardiac calsequestrin gene ( *CASQ2*) • Other genes: *KCNJ2, Ank2,* *TRDN*, and *CALM1*	• Induction of bidirectional or polymorphic VT in the presence of structurally normal heart and ECG (class I) • Diagnosed carriers of a pathogenic mutation on genetic screening ( *RyR2* or *CASQ2*)	• Avoidance of competitive sports, stress environments, and strenuous exercise (class I) • BBs recommended for all patients (class I), genetically positive family members of proband without exercise induced VT (class IIa) • Flecainide should be considered in addition to BBs in patients with recurrent syncope or VT or with contraindication to ICD (class IIa) or in patients with ICD to reduce shocks (class IIa) • ICD and BBs and/or flecainide in patients with SCA, recurrent syncope, or polymorphic VT despite optimal therapy (class I) • LCSD can be performed in CPVT patients with recurrent syncope, arrhythmia, recurrent ICD shocks while on maximal therapy or who are intolerant to medical therapy (class IIb) • Invasive EPS with PVS is not recommended for risk stratification (class III).
HCM	• AD • 10 genes encoding from proteins in cardiac sarcomere • Beta-myosin, troponin T and I, alpha-tropomyosin, myosin light chains, titin, alpha actin, alpha myosin heavy chain, and muscle LIM protein (MLP) • Non-sarcomeric protein mutations (storage disease) clinically similar *PRKAG2, LAMP2* • Commercially available genetic testing	• Left ventricular hypertrophy max wall thickness ≥15 mm, asymmetric (>1.3:1), concomitant obstructive pathology and mitral regurgitation, diastolic dysfunction, abnormal papillary muscles on transthoracic echocardiography or cardiac magnetic resonance imaging (class I) • Genetic testing is recommended in index patient and family members (class I)	• Avoidance of competitive sports, stress environments, and strenuous exercise (class I) • ICD in survivors of SCA or sustained VT/VF causing syncope (class I) • Risk stratification with HCM SCD risk calculator (class I) • BBs or calcium channel blockers (class I) or disopyramide (class IIa) to treat obstructive symptoms. Guideline-directed medical therapy for congestive heart failure (class IIa) • Invasive EPS with PVS is not recommended for risk stratification of SCD (class III)

AD, autosomal dominant; BB, beta blocker; BrS, Brugada syndrome; CPVT, catecholaminergic polymorphic ventricular tachycardia; ECG, electrocardiogram; EPS, electrophysiologic study; ERS, early repolarization syndrome; HCM, hypertrophic cardiomyopathy; ICD, implantable cardioverter defibrillator; LQTS, long QT syndrome; LCSD left cervical sympathetic denervation; PVS, programmed ventricular stimulation; SCA, sudden cardiac arrest; SCD, sudden cardiac death; SQTS, short QT syndrome; VF, ventricular fibrillation; VT, ventricular tachycardia.

An important breakthrough in treating genetic arrhythmia syndromes has been the identification of a potential arrhythmic substrate located in the epicardial right ventricular outflow tract (RVOT) that may trigger VT/VF in some patients with Brugada syndrome. Epicardial 3D EAM has shown abnormal low voltage and fractionated late potentials clustered exclusively in the anterior RVOT epicardium. Ablation at these sites rendered VT/VF non-inducible in 78% and normalization of the Brugada ECG pattern (coved ST-elevations in V1 and V2) in 89%. A multicenter randomized study—Ablation in Brugada Syndrome for the Prevention of VF episodes (BRAVE Study)—is being planned and will assess long-term outcomes of catheter ablation in patients with Brugada syndrome ^38, 39^.

## Medical management of ventricular tachycardia in structural heart disease: Will there be a realm beyond amiodarone therapy?

Pharmacologic therapy for preventing VAs has yielded disappointing results in recent years. Therapy has been limited because of variable efficacy, pro-arrhythmic effects, patient compliance, and adverse effects from long-term therapy. As adjuvant suppressive therapy in patients with ICDs, amiodarone and sotalol have been shown to reduce the rate of recurrent VT (71% and 15–44%, respectively) when compared with beta-blockers or placebo ^40^. Current guidelines recommend pharmacologic therapy (amiodarone or sotalol) with or without adjunctive catheter ablation to prevent VT/VF recurrence and reducing ICD shocks ^8, 16^.

Intravenous amiodarone and sodium channel blockers (lidocaine and procainamide) remain the preferred drug regimen in the acute setting; however, more recently, intravenous sotalol has been shown to terminate sustained VT acutely with a higher efficacy versus lidocaine ^41^. Neither amiodarone nor lidocaine, however, has been shown to improve survival or neurologic outcomes in patients with pulseless VT/VF and out-of-hospital cardiac arrest ^42^. In the first prospective randomized study comparing intravenous procainamide and amiodarone in the acute treatment of hemodynamically tolerated wide complex tachycardia (VAs), procainamide was shown to be more efficacious in tachycardia termination (67% versus 38%) and was associated with fewer major cardiovascular events (9% versus 41%). The effect was consistently observed even in those with structural heart disease as well as when adjusted for age and sex. Although this study had a limited number of patients and was not blinded, it still points to the efficacy and safety of intravenous procainamide in the acute treatment of VT ^43^.

Investigators of the VANISH trial showed an improved composite primary outcome of death, VT storm, or appropriate ICD shocks among patients undergoing catheter ablation versus escalation of anti-arrhythmic drug therapy (amiodarone or mexiletine or both) but with no significant difference between groups in terms of mortality ^44^. In a recent meta-analysis comparing the effectiveness of anti-arrhythmic drugs versus catheter ablation for preventing recurrent VT in patients with ICDs, there was no significant difference between the two treatment modalities (odds ratio (OR) 0.58, 95% confidence interval (CI) 0.26–1.2, *P* = 0.17) in terms of risk reduction of VT as well as all-cause mortality (OR 0.58, 95% CI 0.24–1.24, *P* = 0.23) ^10^. To avoid potential long-term adverse effects and reduce mortality, amiodarone may be safely reduced or discontinued after successful VT ablation without an increase in VT recurrence ^45^.

Nifekalant, a pure potassium channel blocker that has been approved for use in Japan for VT since 1999, has not been shown to be superior to amiodarone in treating out-of-hospital cardiac arrest or shock-resistant sustained VT/VF ^46, 47^. Azimilide, another class III agent (not approved in the US), has been shown to reduce VAs and appropriate ICD shocks in patients with cardiomyopathies, according to data from the SHIELD trial ^48^.

Development of efficacious anti-arrhythmic drugs for VAs with a limited adverse-effect profile continues to be a challenge.

## What lies in the future for implantable device therapy

Next to guideline-directed medical therapy, ICDs can reduce the risk of sudden cardiac death according to several randomized controlled trials for secondary prevention in both ischemic cardiomyopathy and NICM ^11^. For primary prevention of sudden cardiac death, particularly in NICM, the benefit from ICD has been modest primarily because of a lower rate of spontaneous VT/VF. The DANISH investigators ^49^ showed no significant overall mortality benefit from primary prevention ICD implantation in patients with NICM and symptomatic heart failure. Importantly, this observation seems to be age-dependent as patients younger than 59 years derive significant benefit from the ICD.

Intravascular lead-related complications and inappropriate shocks are of particular significance in young patients with channelopathies and syndromes who are also at high risk for fatal VAs. ICD arrhythmia detection and programming algorithms have been refined over time to include higher minimum cutoff rates, extended detection time for VT, anti-tachycardia pacing, and morphology discriminators to minimize the risk of inappropriate shocks ^11, 50^.

The development of an entirely subcutaneous ICD (S-ICD) system with efficacy similar to that of conventional ICD in terminating VT/VF has been a major advance in device technology ^51, 52^. Although randomized comparative data are not yet available, pooled data from the EFFORTLESS and IDE trials have shown the S-ICD to be a safe and effective alternative to conventional transvenous ICDs—especially in younger patients with HCM, genetic channelopathies, and adult complex congenital heart disease—as long as there is no need for pacing ^52– 55^. The rate of inappropriate shocks due to oversensing has been mitigated with the introduction of dual zone programming and algorithms to accommodate R- and T-wave amplitude variations ^56^.

Two additional trials—PRAETORIAN and UNTOUCHED—will provide definitive data regarding a direct comparison of the S-ICD with a transvenous ICD system in terms of efficacy, appropriate and inappropriate shocks, and ICD-related complications ^57, 58^. An investigational percutaneous implantable intravascular defibrillator has also been developed that, compared with conventional ICD, has potentially lower complications and similar defibrillation thresholds ^59^.

## Invasive mapping and catheter ablation of ventricular tachycardia: Have we built the right tools to decode the substrate?

Catheter ablation guided by 3D EAM has emerged as an effective therapeutic option in patients with recurrent and sustained monomorphic VT, arrhythmia-induced cardiomyopathy, recurrent ICD shocks, intolerance to anti-arrhythmic medications, or a combination of these ^16^. Catheter ablation of VT has a reported complication rate ranging from 5 to 7% according to different studies and has low periprocedural mortality and VT termination in up to 70% of cases ^16, 60, 61^. The recurrence rate, however, remains high; 26 to 50% of patients experience recurrent VT during long-term follow-up ^17, 62^.

The success rate of catheter ablation for scar-mediated VT is higher in ischemic cardiomyopathy ^63^. Substrate-guided VT ablation remains the preferred approach particularly when the arrhythmia is poorly tolerated. The VISTA trial showed an extensive substrate-based approach to be superior when compared with targeting clinical VT (15.5% VT recurrence in substrate-guided versus 48.3% in the clinical VT target group) in patients with ischemic cardiomyopathy ^64^. The limitation in achieving higher success in NICM may be related to the more heterogeneous distribution of disease as well as transmurality and epicardial extension of scar ^62^.

Kumar *et al*. ^65^ reported better survival and low VT recurrence in patients without structural heart disease undergoing catheter ablation of VT compared with those with ischemic and non-ischemic cardiomyopathy. During long-term follow-up, the highest VA recurrence was seen in NICM (about 75% of those with NICM had at least one VA recurrence) but patients with ischemic cardiomyopathy had higher overall mortality.

Although we have limited data from randomized controlled trials and no proven mortality benefit yet for VT ablation in structural heart disease, recent studies do suggest that ablation results in improved quality of life, freedom from ICD shocks, and improved transplant-free survival. Tung *et al*. ^66^ reported 1-year freedom from VT recurrence in up to 70% of patients with structural heart disease who underwent VT ablation (including 72% of patients with ischemic cardiomyopathy and 68% with NICM). Lack of recurrent VT translated as an improved transplant-free survival in this study ^66^. Data from the THERMOCOOL VT trial looking at the long-term success of open irrigated radiofrequency (RF) catheter ablation of monomorphic VT associated with coronary artery disease showed reduced ICD shocks and VT episodes with improved quality of life at 6 months. At 3-year follow-up, non-recurrence resulted in decreased amiodarone use and hospitalizations ^67^.

Other investigational and clinically tested techniques that could potentially improve substrate characterization, procedural outcomes and minimize complications include use of irrigated and contact force ablation catheters, incorporation of pre-procedural and intra-procedural imaging, ablation of deep myocardial substrate with bipolar RF ablation, transcoronary alcohol injection, coronary coil embolization, epicardial ablation, surgical cryoablation, and remote magnetic navigation, and stereotactic ablative radiotherapy ( Table 2) ^68– 78^. However, concrete data on the efficacy of these techniques is lacking and further studies are needed.

**Table 2.** Investigational modalities for ventricular tachycardia ablation and suggested clinical applications.

Innovative modality	Investigators and initial reports	Salient features and possible benefits
MRI-guided stereotactic ablative radiotherapy • For patients with VT who have failed standard therapy • Uses anatomic scar imaging: MRI or SPECT or both with superimposed voltage map to accurately delineate scar	Cuculich *et al*. - ENCORE-VT phase I/II study ^83^ Clinical trial: NCT02919618 Loo *et al*. Stereotactic Ablative Radiotherapy (STAR) ^84^ Maguire *et al*. - CyberHeart’s Cardiac Arrhythmia Ablation ^85^ Clinical trial: NCT02661048	• A rapid and totally non-invasive technique • Pre-clinical data suggest significant reduction in VT burden (>50%) and reduction in implantable cardioverter defibrillator therapy (>85%). • Clinical trials to determine the acute and long-term safety and efficacy initiated • Gap-free ablation can be performed in short duration for medically frail population
Infusion needle catheter ablation • Infusion needle catheter ablation is being studied for effectiveness in patients who have failed standard RF ablation, especially with deep mid-myocardial substrate for VT • Needle design allows for myocardial penetration, infusion of heated saline and RF energy delivery • Pre- and peri-procedural imaging helps guide the procedure (e.g; intracardiac echocardiography)	Sapp *et al*. ^77^ Stevenson *et al*. Intramural Needle Ablation for Recurrent Ventricle Tachycardia ^86^ Clinical trial: NCT01791543	• A bipolar or quadripolar catheter equipped with an extensible needle that can project up to 12 mm beyond tip is used • Heated saline can be infused through tip and side pores • Flow rate of saline determines lesion size • Large lesions can be created, does not require scar/lesion maturity
Facilitated RF ablation using magnetically directed nanoparticles • Iron, carbon, or titanium nanoparticles are delivered within heat-sensitive liposomes • Iron nanoparticles used in imaging considered safe • Study *in vivo* porcine models	Nguyen *et al*. ^87^	• Nanoparticles have been shown to alter tissue sensitivity to RF energy with a potential to create deeper lesions • Better tissue heating avoids “steam pops”
MRI-based signal intensity mapping for epicardial substrate • The technique helps to identify epicardial substrate for monomorphic VT with contrast enhanced MRI • Images can be correlated with standard electroanatomical mapping • Clinical data based on *in vivo* porcine models available	Arenal *et al*. ^33^	• Epicardial VT substrate can be identified by contrast-enhanced MRI-based signal intensity mapping • Patchy scar pattern may be associated with VT inducibility • Potential non-invasive imaging modality to determine risk of inducible epicardial VT prior to planned procedure
MIBG imaging to guide VT ablation • MIBG has characteristics similar to those of norepinephrine; it is taken up at presynaptic neurons • 3D models with MIBG are created to guide VT ablation • A 15-patient feasibility study • ADMIRE trial identified areas of cardiac denervation and correlations with death, heart failure, and VT/ventricular fibrillation • MIBG (scan) US Food and Drug Administration-approved 2013	Klein *et al*. - 3D MIBG Innervation Maps to assess substrate and successful ablation sites for VT ^88^ Jacobson *et al*. ADMIRE-HF ^89^	• MIBG innervation defects are larger than scars created with bipolar voltage maps. • In the pilot study 36% successful ablative sites were located in areas of abnormal innervation and normal voltage • Voltage maps do not necessarily correlate with innervation maps. • Innervation maps may help target additional sites of VT ablation not identified by EAM
Non-invasive high-resolution mapping and ECGI • This technique offers single-beat panoramic mapping • Both endocardial and epicardial mapping can be performed	Reports from 1998 to 2015; Rudy *et al*. ^90, 91^ Zhang *et al*. ^92^	• Non-invasive modality to provide endocardial and epicardial substrate information • Information can be used to risk-stratify patients and identify those at highest risk of ventricular arrhythmia. • ECGI has been shown to have a high correlation with MRI and SPECT derived scar • Anti-arrhythmic drug exposure could potentially affect amplitude and fractionation.
Cardiac ripple mapping for slow conducting channels • A novel method to incorporate voltage and activation data for EAM mapping • High-density bipolar electrograms collected	Luther *et al*. ^93^ Jamil-Copley *et al*. ^94^	• Allows for simultaneous visualization of voltage and activation data incorporated on 3D EAM • Enables identification of slow conduction channels within scar zones in myocardium that may be potential targets of VT ablation • Target-specific approach can improve efficacy of ablation.

3D, three-dimensional; EAM, electroanatomic map; ECGI, electrocardiographic imaging; MIBG, ^123^I-meta-iodobenzylguanidine; MRI, magnetic resonance imaging; RF, radiofrequency; SPECT, single-photon emission computed tomography; VT, ventricular tachycardia.

However, concrete data for their efficacy are lacking and more studies are needed. Epicardial substrate ablation has been identified as a potentially successful ablation strategy to eliminate VAs in select patients with ARVD and NICM and more recently in patients with Brugada syndrome ^38, 39, 79^. In patients with incessant VT who have failed conventional and ablative therapy, left or bilateral cervical sympathectomy has been shown in observational studies to reduce VT recurrence, but larger studies are needed to establish the safety and efficacy of this procedure ^80– 82^.

## Conclusions

VAs encompass a broad range of clinical conditions ranging from benign to life-threatening. Sustained VAs are a major cause of morbidity and mortality in patients with structural heart disease. In addition to refinement and evolution of well-established therapies such as anti-arrhythmic drugs and ICDs, several major advances in the mechanistic, genetic, diagnostic, and therapeutic realms have furthered our understanding of the pathophysiology of ventricular tachyarrhythmias and helped fashion safer and more effective therapies particularly for drug-refractory VAs. Several gaps in our knowledge remain and require further research. Ultimately, successful short- and long-term management of VAs will likely integrate biological, pharmacologic, device-based, and ablative therapies.

## Abbreviations

3D, three-dimensional; ARVD, arrhythmogenic right ventricular dysplasia; CI, confidence interval; CMR, cardiac magnetic resonance imaging; CT, computed tomography; EAM, electroanatomic mapping; ECG, electrocardiogram; HCM, hypertrophic cardiomyopathy; ICD, implantable cardioverter defibrillator; MRI, magnetic resonance imaging; NICM, non-ischemic cardiomyopathy; OR, odds ratio; RF, radiofrequency; RVOT, right ventricular outflow tract; S-ICD, subcutaneous ICD; VA, ventricular arrhythmia; VF, ventricular fibrillation; VT, ventricular tachycardia.
